# Prevalence of birth before arrival and associated factors among postpartum women in southern Ethiopia: a community-based cross-sectional study

**DOI:** 10.3389/fmed.2024.1437538

**Published:** 2024-09-27

**Authors:** Tsedeke Amanuel, Mitiku Desalegn, Kaleegziabher Lukas, Tadele Yohannes

**Affiliations:** ^1^Lemo Woreda, Health Bureau, Hosanna, Ethiopia; ^2^Department of Anesthesia, College of Medicine and Health Science, Wachemo University, Hosaina, Ethiopia; ^3^School of Public Health, College of Medicine and Health Sciences, Wachemo University, Hosaina, Ethiopia

**Keywords:** birth before arrival, prevalence, associated factors, southern Ethiopia, women

## Abstract

**Background:**

Birth before arrival (BBA) constitutes a high-risk newborn population with high perinatal morbidity and mortality. In Ethiopia, most studies and health surveys consider only home and hospital deliveries but do not consider deliveries that take place between the house and health facility. The aim of this study was to assess the prevalence of BBA and its associated factors among postpartum women in Lemo woreda, Hadiya Zone, SNNPR, Ethiopia, 2023.

**Methods:**

A community-based cross-sectional study was conducted among postpartum women in Lemo woreda, Hadiya Zone, SNNPR, Ethiopia, from April 5 to May 20, 2023. Three hundred eighty-two postpartum women who gave birth 6 months prior to this study were included. Twelve out of 36 kebeles were selected randomly, and simple random sampling was employed for the selection of participant women. An interviewer-administered questionnaire was used for data collection. A binary logistic regression analysis was computed, and variables with a *p* value of <0.25 were included in the final multivariable logistic regression analysis. Model fitness was checked via the Hosmer–Lemeshow goodness-of-fit test (*x*^2^ = 16.04, *p* value = 0.250). Statistical significance was declared via odds ratios and 95% confidence intervals at a *p* value <0.05.

**Results:**

The prevalence of BBA among women who gave birth in the last 6 months preceding this study in the study area was 15.2% (95% CI: 11.8, 19.1%). In the multivariable analysis, the variables associated with birth before arrival in the final model were having no antenatal care (AOR = 2.63; 95% CI: 1.23, 5.63), having a female autonomy status (AOR = 3.32; 95% CI: 1.12, 9.89), not being knowledgeable about labor symptoms (AOR = 2.15; 95% CI: 1.11, 4.18), and having birth preparedness toward the index birth (AOR = 0.13; 95% CI: 0.05, 0.35).

**Conclusion:**

The prevalence of BBA in the study area was unacceptably high. A statistically significant association was observed between birth before arrival and having no antenatal care, dependent women’s autonomy status, being not knowledgeable about labor symptoms, and having birth preparedness toward the index birth.

## Introduction

Births before arrival (BBA) at a health facility are defined as unplanned deliveries before arrival at a health institution (1). These births outside of health facilities can occur on the way to the hospital or in ambulances and are not attended by a midwife or medical officer (2, 3). BBA is a preventable phenomenon, but it is still common in modern-day practice (4) despite the wide-ranging progress made toward accessibility for obstetric care in Ethiopia. The prevalence rate of BBA in developed countries is less than 1% (5, 6), which increases exponentially in low-income countries to greater than 50% in countries such as India and Ethiopia (1, 7). The rate of BBA in developed country is similar from one hospital setting to other. The rate in England is reported as 0.5% (8), which is similar with Australia (9) and Ireland (10), whereas in the United States the rate was higher at 1.4% (11). However the incidence of BBA in developing country is higher although there is a paucity of data. In South Africa, in 2009, an incidence rate of 5.7% in KwaZulu-Natal (KZN) and 5% in Gauteng was reported by the National Department of Health (1). According to the Mini Ethiopian Demographic and Health Survey (EDHS) 2019 report, 48% of the total live births in the 5 years preceding the survey were delivered in a health facility, and skilled delivery attendance in the Hadiya Zone of southern Ethiopia was 78% in 2018 (7, 12). The rate of BBA serves as an index of accessibility to perinatal care; a rate greater than 1.5% signals challenges in healthcare provision (13).

BBA is associated with unfavorable perinatal outcomes and increased maternal morbidity and mortality (14, 15). The perinatal morbidities associated with BBA include hypothermia, asphyxia, respiratory distress, low birth weight, neonatal sepsis, and an increased rate of mother–child transmission of the human immunodeficiency virus (HIV) (16, 17), among which hypothermia is the most prevalent in newborns (14, 18, 19). Neonatal mortality (NM) in Ethiopia decreased from 39 to 29 per 1,000 live births between the 2005 and 2016 EDHS but has remained stable since the 2016 EDHS, and currently, it is 30 per 1,000 live births according to the 2019 mini EDHS (7).

Many women continue to give birth outside of health facilities without skilled attendance, with the risk of severe complications and mortality (3). Maternal mortality (MM) is highest in sub-Saharan Africa (SSA) and Southeast Asia, which together account for 85% of the global burden (3, 20). Maternal complications resulting from having a BBA include postpartum hemorrhage due to uterine atony, retained placenta and products of conception, obstetrical lacerations and tears, and puerperal sepsis (17). Given the increasing NM and unpromising reduction in MM in Ethiopia, where the BBA is the contributing factor, identification of its associated factors is needed.

Some studies from Africa have demonstrated that sociodemographic factors, such as low maternal age, low educational level, rural residence, marital status, and monthly income; obstetric factors, such as poor antenatal attendance, unwanted and teenage pregnancies, mode of previous delivery, birth plans, and recognition of the onset of labor; and access factors, such as poor access to transport and health care, are associated with BBA (2, 9, 16, 21–23). Health facility intrapartum strategies, with a skilled birth attendant and efficient referral of pregnancy and childbirth complications, have been shown to be the most effective in addressing maternal and early neonatal mortality (3, 24). The government of the Federal Democratic Republic of Ethiopia and its stakeholders have made tremendous efforts, such as increasing health service accessibility and coverage, providing maternal health services, and providing postnatal care follow-up free of charge (25), to address births in out-of-health facilities and consequently reduce child and maternal mortality. Despite these efforts, only 48% of women give birth at health facilities in Ethiopia.

Much of the available literature surrounding the BBA is from developed and middle-income countries, and the author found no recent evidence from Ethiopia. According to available evidence the prevalence of BBA in developing country is higher which is associated with multiple maternal and neonatal complications. There is no data in Ethiopian context about its prevalence and associated factor, but it’s presumed to be higher. Furthermore, those available studies include intentional and planned home deliveries as BBAs, which could increase their prevalence. Most of these studies were facility-based and conducted in urban settings and focused on the effect of BBA on adverse perinatal outcomes where its associated factors were not adequately addressed. The primary objective of this study was to determine the prevalence of BBA and the secondary objective is to identify risk factors for BBA in the study area. We aimed to build a model for the prediction of BBA to ascertain if factors identified may be used to assist clinical decision-making and risk stratification at the earliest stages of pregnancy. Thus, the current study aimed to determine the prevalence of BBA and their associated factors, in Lemo woreda, Hadiya Zone.

## Methods and materials

### Study area and period

The study was conducted in Lemo woreda, which is one of the 13 districts and four town administrations of the Hadiya Zone in southern nations, nationalities, and people’s regional states. The estimated total population of the woreda in 2015 E.C. was 170,948 (84,722 males and 86,226 females) and 39,831 childbearing-aged women, with an estimated 3.46% pregnant women. Lemo woreda has 36 kebeles, of which 33 are rural kebeles and 3 are urban kebeles. There are 33 rural health posts and 34,887 households in the woreda. The total number of live births in Lemo woreda in 2022 was 5,915 (12). The woreda is located 230 kilometers southwest of Addis Ababa, the capital of Ethiopia. The study was conducted from April 5 to May 20, 2023.

### Participants

A community-based cross-sectional study was conducted among 382 postpartum women who gave birth in the last 6 months before the current study and who reside in Lemo Woredas. Postpartum women who were not able to respond to the study questionnaires due to their health status were excluded from the study.

### Sample size and sampling technique

The sample size was determined via the use of Epi-Info version 7.2.2.6 software for primary objective (prevalence of BBA) estimation via sample size estimation for a single population and with the following assumptions: confidence level of 95%, proportion of 60% delivery out of health facilities in rural areas of Ethiopia from mini EDHS 2019 (7), population size of 5,915 and a 5% margin of error. Considering a 10% nonresponse rate, the final minimum sample size calculated was 347 + 10% nonresponse rate, which is 382. Sample size calculation for the secondary objective (associated factors of BBA) was performed via Epi-Info Statcalc version 7.2.2.6 via the following formula for a cross-sectional study: 95% confidence interval, 80% power, 1:1 exposure-to-nonexposure ratio, and a design effect of 1.0 (Table 1).

**Table 1:** Sample size calculation for associated factors of BBA (22).

Factors	Category	AOR	P_0_	Calculated sample size		
				Exposed	Unexposed	Total
Marital status	Married	1				
	Unmarried	1.87	43.0%	175	175	350
Residence	Rural	1				
	Urban	0.39	90.0%	158	158	316
Wealth index	1-poorest	1				
	2	0.46	75.0%	132	132	264
	3	0.51	61.0%	153	153	306
	4-least poor	0.48	73.0%	142	142	284

P_0_: % outcome in the unexposed group; AOR: adjusted odds ratio.

Therefore, the final sample size used in this study was 382, which was calculated for the first objective because it yields the largest sample size compared with the secondary objective. Thirty percent of the kebeles, on the basis of the rule of thumb, were taken.

### Sampling procedure

**Figure 1:**
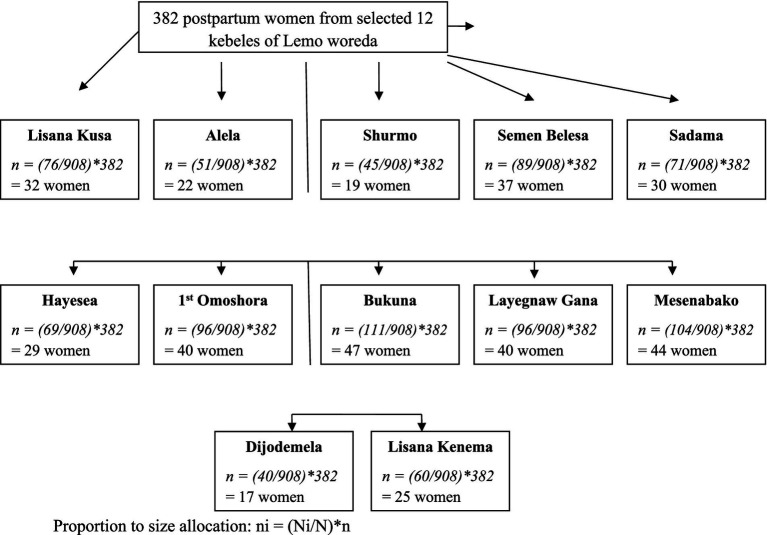
Schematic representation of the sampling procedure indicating the proportional allocation of sample size to selected kebeles in Lemo woreda, 2023.

The last 6 months’ delivery report of the selected kebeles for total deliveries during the period (October 1st to March 31st, 2015 E.C.) was taken from the health extension master family index registry. These included 908 postpartum women in 12 selected kebeles and were used to proportionally allocate the sample size to each selected kebee. A simple random sampling technique was used based on random number generation to select postpartum women from the sampling frame, which is the list of postpartum women who gave birth during the last 6 months from the selected kebeles (see Figure 1).

### Study variables

#### Dependent variable: BBA

##### Independent variables

*Socio demographic factors* (age, residence, educational level, marital status, maternal occupation, husband occupation, husband education, monthly income, media exposure, and housing characteristics), *reproductive health and obstetric characteristics* (gravidity, parity, contraceptive use, pregnancy type, ANC follow-up, duration of labor, mode of previous delivery, recognition of onset of delivery, knowledge of EDD, birth preparedness and emergency readiness plan, and women’s autonomy), and *access to health facilities* [maternal delay (1st and 2nd), means of transport, and time of delivery].

##### Operational definitions

###### Birth before arrival

A woman was classified as having had a birth before arrival if she delivered outside the health facility either en route to the health care facility or in ambulances and was not attended to by a skilled birth attendant.

###### Skilled birth attendance

When the delivery was attended by an obstetrician, a general practitioner, a public health officer, a midwife, a nurse, or a level IV health extension worker.

###### The first delay

The first delay (delay in seeking health care) was measured in hours and was categorized into <24 h and ≥ 24 h (26).

###### The second delay

The second delay (delay to reach the health facility) was measured in the travel time it took to walk to the facility on foot, and it was categorized as <2 and ≥ 2 h according to the Ethiopian travel time standard (27).

###### Birth preparedness

Respondents who had at least one of the components of the birth preparedness plan in their current pregnancy were considered to have birth preparedness and otherwise no birth preparedness.

###### Women’s autonomy

Women’s autonomy was assessed by asking questions about who in the household makes decisions regarding maternal health care, large household purchases, visits to family and relatives, and child health care. For each of the four questions, a respondent received 1 point if she was involved in the decision and 0 points if she was not. These points were summed to yield total scores ranging from 0 to 4 (28). The individual respondent’s score was obtained by dividing the score by the maximum score, which was 4. A score > 0.5 was considered autonomous and otherwise unautonomous or dependent. The average value of the women’s autonomy index was calculated by adding up individual scores and dividing by the number of respondents.

###### Knowledge of labor symptoms

Respondents who answered more than half of the knowledge questions were considered to have good knowledge or otherwise poor knowledge. Each respondent was asked five knowledge questions regarding signs and symptoms of labor: strong and frequent contractions, belly and back pain, a bloody show, water breaks, and the urge to use a toilet.

### Data collection techniques and instruments

Data were collected via a structured interviewer-administered questionnaire, which was developed after a thorough review of the literature. It includes sociodemographic, reproductive health, and obstetric characteristics, as well as access to health facility parts. A face–to-face interview was held to gather data. Trained health extension workers working in kebeles collected the data. The interviews were held in a private area at the women’s homes. The overall data collection process was supervised by trained midwives working in the study area. The information was collected during a retrospective birth history, in which female respondents provided a history of delivery.

### Data processing and analysis

Each questionnaire was checked for completeness, coded, and entered into Epi-Data Version 4.4 and exported to SPSS for Windows version 24 for analysis. The analysis was performed after data cleaning. Frequencies, proportions, and measures of variation were used to describe the study population in terms of sociodemographics and other relevant variables. A binary logistic regression model was constructed. Binary logistic regression was used to determine the association between each independent variable and the outcome variable, and a *p* value of <0.25 was used to recruit variables for the final multivariable logistic regression model. Model fitness was checked via the Hosmer–Lemeshow goodness-of-fit test (*x*^2^ = 16.04, *p* value = 0.250). Statistical significance was determined via odds ratios (ORs) and 95% confidence intervals (CIs) at *p* values <0.05 in multivariable logistic regression.

### Data quality management

The questions prepared in English were translated into Hadiyssa and back-translated to English by different expert translators to check for consistency. A pretest was carried out at Ashe Kebele on 5% of the sample size (19 women) for 1 week. Any inconsistencies and misspellings that affect contextual understanding were corrected in the final Hadiyssa version of the questionnaire. Data collectors were trained for 2 days on the objectives of the study, data collection techniques and tools, and data consistency and completeness were checked daily by a trained supervisor and the principal investigator, and spot corrections were taken. After the data were collected, each questionnaire was coded, and data cleaning was performed before actual data analysis.

## Results

### Sociodemographic characteristics

A total of 382 study participants were included in the analysis, with a 100% response rate. The mean age of the participants was 30.3 ± 4.4 years. The majority of the participant women were in the 23–26 years age group (28.3%), but women in the 27–30 years age group were the most likely to have experienced birth before arrival (34.6%), and three-fourths of them were rural residents (76.2%). Nearly half of the women (45.5%) had attained secondary education (grades 9–12). The majority of the participant mothers (83.8%) were housewives in their occupation, whereas 68.0% of their husbands were farmers. The mean estimated monthly income of the family was 7,216 birr ±3,073.4 SD. The majority of participants (95.0%) had media exposure, and radio was the most common medium (Table 2).

**Table 2:** Sociodemographic characteristics of women who gave birth in the last 6 months in Lemo district, Hadiya zone, southern Ethiopia (*n* = 382).

Variables	Category	Frequency (percentage)		Total
		No BBA	BBA	
Mother’s age in years Range (23–38)	23–26	105 (97.2%)	3 (2.8%)	108 (28.3%)
	27–30	70 (65.4%)	37 (34.6%)	107 (28.0%)
	31–34	78 (88.6%)	10 (11.4%)	88 (23.0%)
	35–38	71 (88.9%)	8 (10.1%)	79 (20.7%)
Residence	Urban	74 (81.3%)	17 (18.7%)	91 (23.8%)
	Rural	250 (85.9%)	41 (14.1%)	291 (76.2%)
Women’s educational status	No formal education	31 (60.8%)	20 (39.2%)	51 (13.4%)
	Primary	154 (88.5%)	20 (11.5%)	174 (45.5%)
	Secondary	126 (89.4%)	15 (10.6%)	141 (36.9%)
	Higher education	13 (81.3%)	3 (18.7%)	16 (4.2%)
Husband’s educational status	No formal education	5 (41.7%)	7 (58.3%)	12 (3.1%)
	Primary	132 (89.8%)	15 (10.2%)	147 (38.5%)
	Secondary	160 (86.5%)	25 (13.5%)	185 (48.4%)
	Higher education	27 (71.1%)	11 (28.9%)	38 (10.0%)
Women’s occupation	Housewife	272 (85.0%)	48 (15.0%)	320 (83.8%)
	Maid	38 (86.4%)	6 (13.6%)	44 (11.5%)
	Civil servant	14 (77.8%)	4 (22.2%)	18 (4.7%)
Husband’s occupation	Farmer	229 (88.1%)	31 (11.9%)	260 (68.0%)
	Merchant	80 (77.7%)	23 (22.3%)	103 (27.0%)
	Civil servant	15 (78.9%)	4 (21.1%)	19 (5.0%)
Housing characteristics	Private	295 (85.3%)	51 (14.7%)	346 (90.6%)
	Rented	29 (80.6%)	7 (19.4%)	36 (9.4%)
The average estimated monthly income of the family in Birr	2,000-6,000	162 (89.0%)	20 (11.0%)	182 (47.6%)
	6,001-11,000	126 (78.7%)	34 (21.3%)	160 (41.9%)
	11,001-15,000	36 (90.0%)	4 (10.0%)	40 (10.5%)
Media exposure	Yes	310 (85.4%)	53 (14.6%)	363 (95.0%)
	No	14 (73.7%)	5 (26.3%)	19 (5.0%)
Type of media (*n* = 363)	Radio	277 (88.8%)	35 (11.2%)	312 (81.7%)
	Television	28 (65.1%)	15 (34.9%)	43 (11.2%)
	Social media	5 (62.5%)	3 (37.5%)	8 (2.1%)

BBA, Birth before arrival. birr, Ethiopian currency.

### Reproductive health and obstetric characteristics

The majority of the participating women (76.7%) had antenatal care (ANC) follow-up for their last pregnancy. Two-thirds of women had 1--3 ANC contacts for their last delivery, with a mean of 1.2 ± 0.4 SD. The proportion of women who used contraceptives before their last pregnancy was 26.4%. Two-thirds of the participant women (71.7%) had postnatal care (PNC) follow-up for their last birth, and 76.2% of the women had planned and wanted this type of last pregnancy. The average value of the women’s autonomy index was 0.68, and 26.7% of the participants were autonomous. The proportion of women with grand multigravida was 21.0%, the duration of labor of index birth ≥12 h was 54.7%, birth preparedness toward index birth was 86.1%, women with knowledge of the expected date of delivery (EDD) of the index birth were 66.7, and 66.1% of women knew labor symptoms. The mean number of pregnancies was 3.0 ± 1.6, and the median duration of labor for the last birth was 14 (IQR 12–24; Table 3).

**Table 3:** Reproductive health and obstetric characteristics of participant women by birth before arrival in Lemo district, Hadiya zone, southern Ethiopia (*n* = 382).

Variables	Category	Frequency (percentage)		Total
		No BBA	BBA	
ANC for the last delivery	Yes	254 (86.7%)	39 (13.3%)	293 (76.7%)
	No	70 (78.7%)	19 (21.3%)	89 (23.3%)
Number of ANC contacts (*n* = 293)	1–3 contacts	178 (86.0%)	29 (14.0%)	207 (70.6%)
	≥ 4 contacts	76 (88.4%)	10 (11.6%)	86 (29.4%)
Type of health facility for ANC (*n* = 293)	At hospital	14 (82.4%)	3 (17.6%)	17 (5.8%)
	At health center	177 (87.2%)	26 (12.8%)	203 (69.3%)
	At health post	63 (86.3%)	10 (13.7%)	73 (24.9%)
Used contraceptive before last pregnancy	Yes	83 (82.2%)	18 (17.8%)	101 (26.4%)
	No	241 (85.8%)	40 (14.2%)	281 (73.6%)
PNC for the last delivery	Yes	233 (85.0%)	41 (15.0%)	274 (71.7%)
	No	91 (84.3%)	17 (15.7%)	108 (28.3%)
Type of pregnancy	Planned and wanted	249 (85.6%)	42 (14.4%)	291 (76.2%)
	Unplanned but wanted	68 (91.9%)	6 (8.1%)	74 (19.4%)
	Unplanned and unwanted	7 (41.2%)	10 (58.8%)	17 (4.4%)
Women autonomy	Nonautonomous	231 (82.5%)	49 (17.5%)	280 (73.3%)
	Autonomous	93 (91.2%)	9 (8.8%)	102 (26.7%)
Gravidity	Multigravidae	259 (85.8%)	43 (14.2%)	302 (79.0%)
	Grand multigravidae	65 (81.3%)	15 (18.7%)	80 (21.0%)
Duration of labor of index birth	< 12 h	155 (89.6%)	18 (10.4%)	173 (45.3%)
	≥ 12 h	169 (80.9%)	40 (19.1%)	209 (54.7%)
Know the EDD of the index birth	Yes	226 (88.6%)	29 (11.4%)	255 (66.7%)
	No	98 (77.2%)	29 (22.8%)	127 (33.3%)
Birth preparedness toward index birth	Yes	285 (86.6%)	44 (13.4%)	329 (86.1%)
	No	39 (73.6%)	14 (26.4%)	53 (13.9%)
Knowledge of labor symptoms (*n* = 350)	Not knowledgeable	89 (74.8%)	30 (25.2%)	119 (34.0%)
	Knowledgeable	205 (88.7%)	26 (11.3%)	231 (66.0%)
Mode of previous delivery	SVD	217 (85.1%)	38 (14.9%)	255 (66.7%)
	Instrumental delivery	34 (94.4%)	2 (5.6%)	36 (9.4%)
	SVD with episiotomy	72 (80.9%)	17 (19.1%)	89 (23.3%)
	Cesarean section	1 (50.0%)	1 (50.0%)	2 (0.6%)

ANC, antenatal care; BBA, birth before arrival; EDD, expected date of delivery; PNC, postnatal care; SVD, spontaneous vaginal delivery.

### Access to a healthcare facility

The median time to seek health care was 14 h (IQR 12–24), and the mean duration of the second delay was 1.3 h ± 0.6 SD. One-fourth (25.9%) of women delayed ≥24 h in deciding to seek health care, and one-third (33.8%) traveled ≥2 h to reach a health care facility. The major reason for the first delay was not realizing the problem (28.8%) for women with birth before arrival, and lack of transport (62.3%) was the major reason for the second delay. As a means of transport, 35.8% of women used horse or donkey carts. Regarding the condition of the road, 29.3% of women replied that it is a wide road. More than half of the women (51.6%) delivered their index birth at night (Table 4).

**Table 4:** Access to health care facilities among women who gave birth in the last 6 months in Lemo district, Hadiya zone, southern Ethiopia (*n* = 382).

Variables	Category	Frequency (percentage)		Total
		No BBA	BBA	
First delay	Delayed <24 h	246 (86.9%)	37 (13.1%)	283 (74.1%)
	Delayed ≥24 h	78 (78.8%)	21 (21.2%)	99 (25.9%)
Second delay (*n* = 317)	Traveled <2 h	171 (81.4%)	39 (18.6%)	210 (66.2%)
	Traveled ≥2 h	88 (82.2%)	19 (17.8%)	107 (33.8%)
Reasons for 1st delay	Underestimate severity	195 (86.7%)	30 (13.3%)	225 (100%)
	Did not realize the problem	116 (71.2%)	47 (28.8%)	163 (100%)
	Essential person for decision making not around	78 (86.7%)	12 (13.3%)	90 (100%)
Reasons for 2nd delay (*n* = 317)	Lack of money	61 (87.1%)	9 (12.9%)	70 (22.1%)
	Lack of transport	159 (80.7%)	38 (19.3%)	197 (62.3%)
	Distant health facility	38 (77.6%)	11 (22.4%)	49 (15.6%)
Means of transport (*n* = 317)	Motorbike	39 (100.0%)	0 (0.0%)	39 (12.3%)
	Ambulance	16 (48.5%)	17 (51.5%)	33 (10.4%)
	Public transport	62 (100.0%)	0 (0.0%)	62 (19.6%)
	Horse/Donkey cart	83 (73.5%)	30 (26.5%)	113 (35.8%)
	On foot	14 (58.3%)	10 (41.3%)	24 (7.6%)
	Carried by other people	44 (97.8%)	1 (2.2%)	45 (14.2%)
Status of the road	All weather road	225 (83.3%)	45 (16.7%)	270 (70.7%)
	Weather road	99 (88.4%)	13 (11.6%)	112 (29.3%)
Time of delivery of index birth	Night	150 (76.1%)	47 (23.9%)	197 (51.6%)
	Day	174 (94.1%)	11 (5.9%)	185 (48.4%)

Women who delivered their last birth at home were not asked about the second delay or its reasons.

### The primary objective: prevalence of BBA

The prevalence of birth before arrival among women who gave birth in the last 6 months preceding this study in the study area was 15.2% (95% CI: 11.8, 19.1%). Among them, 84.5% of women gave birth on the route to a health care facility (Table 5).

**Table 5:** Profile of births before arrival among women who gave birth in the last 6 months in Lemo district, Hadiya zone, southern Ethiopia (*n* = 382).

Variables	Frequency	Percentage
Place of last delivery		
Home	67	17.5
Before arrival to a health care facility	58	15.2
Health care facility	257	67.3
The specific place of BBA to health care facility (*n* = 58)		
On the route to health care facility	49	84.5
In ambulance	9	15.5

BBA, Birth before arrival.

### The secondary objective: factors associated with BBA

Using bivariate binary logistic regression analyses, variables with a *p* value of <0.25 were included in the final model. Thus, in the multivariable analysis, the variables found to be associated with birth before arrival in the final model were having no antenatal care (AOR = 2.63; 95% CI: 1.23, 5.63), dependent women’s autonomy status (AOR = 3.32; 95% CI: 1.12, 9.89), being not knowledgeable about labor symptoms (AOR = 2.15; 95% CI: 1.11, 4.18), and having birth preparedness toward index birth (AOR = 0.13; 95% CI: 0.05, 0.35).

Therefore, this study revealed that women who had no antenatal care visits during the index pregnancy had three times greater odds of birth before arrival than women with antenatal care. The probability of having a birth before arrival at a health care facility was three times greater for women who were dependent on their autonomy status than for those who were autonomous. Moreover, those women who were not knowledgeable about labor symptoms were two times more likely to experience birth before arrival than those who were knowledgeable. Compared with women with no birth preparedness plan, those with birth preparedness toward the index pregnancy were 87% less likely to experience birth before arrival. Having first and second delays, status of the road, time of delivery, and income were unrelated to the probability of birth before arrival (Table 6).

**Table 6:** Multivariate logistic regression showing factors associated with BBA among women who gave birth in the last 6 months in Lemo District, Hadiya Zone, southern Ethiopia.

Variables	Category	COR (95% CI)	AOR (95% CI)	*p*- value
Media exposure	Yes	1.00	1.00	0.103
	No	2.09 (0.72, 6.04)	2.74 (0.82, 9.18)	
Receive ANC in last pregnancy	Yes	1.00	1.00	0.013
	No	1.77 (0.96, 3.25)	2.63 (1.23, 5.63)	
Women autonomy	Unautonomous	2.19 (1.04, 4.64)	3.32 (1.12, 9.89)	0.031
	Autonomous	1.00	1.00	
Knowledge of labor symptoms	Not knowledgeable	2.66 (1.49, 4.75)	2.15 (1.11, 4.18)	0.023
	Knowledgeable	1.00	1.00	
Birth preparedness for the last birth	Yes	0.43 (0.22, 0.86)	0.13 (0.05, 0.35)	< 0.001
	No	1.00	1.00	
Used contraceptive before last pregnancy	Yes	1.31 (0.71, 2.40)	1.70 (0.78, 3.69)	0.181
	No	1.00	1.00	
First delay	Delayed <24 h	1.00	1.00	0.082
	Delayed ≥24 h	1.79 (1.00, 3.24)	1.86 (0.92, 3.76)	

ANC, antenatal care; AOR, adjusted odds ratio; CI, confidence interval; COR, crude odds ratio.

## Discussion

The prevalence of BBA among women who gave birth in the last six months preceding this study in the study area was unacceptably high. In the current study, the variables associated with BBA in the Lemo district of the Hadiya zone were having no antenatal care, being dependent on women’s autonomy status, being not knowledgeable about labor symptoms, and having birth preparedness toward the index birth.

Globally, the prevalence of BBA at a healthcare facility is estimated to be less than 1% of all deliveries in developed countries (1, 5, 14, 18, 19). However, the prevalence of BBAs has increased exponentially in low-income countries to greater than 50% (1, 7). In the present study, the prevalence of BBA was 15.2%. This is a high prevalence in the study area, as the rate of BBA is used as an index of accessibility to perinatal care, and a rate greater than 1.5% signals challenges in health care provision where appropriate interventions are praiseworthy (13). Births occurring between home and health care facilities, either en route to health care facilities or in ambulances, were given less attention, and no recent evidence was found in Ethiopia that indicated their prevalence. However, according to the mini-EDHS 2019 report, 40% of the total live births were delivered in health facilities in rural areas, 58.7% were delivered at home, and the gap of 1.2% reported might be due to the prevalence of BBAs in health facilities, even though it has not been reported (7).

The findings of the present study were greater than those reported by the EDHS, and this difference might be due to misclassification bias. Accordingly, in the EDHS questionnaire, there was no option indicated for BBA, so the BBA prevalence might be misclassified as home or health facility delivery prevalence, but in the current study, the option for BBA at a health facility was indicated in the questionnaire. However, the prevalence of BBA in South Africa is 5.4%, and in rural Malawi, it is 9% (4, 22). According to the findings of the present study, the prevalence of BBAs in Lemo district was greater than the prevalence in South Africa and rural Malawi. The difference might be due to the variation in socioeconomic status across countries.

This study revealed that women who had no antenatal care visits during the index pregnancy had three times greater odds BBA than women with antenatal care. Similarly, a national register study in Finland revealed that one of the predictors of deliveries before arrival at a health care facility was fewer prenatal visits (29). Another study from Tharaka Nithi County, Kenya, reported that the obstetric risk factors associated with BBA were ANC attendance, the timing of ANC attendance, and the number of ANC visits (23). A prospective case–control study from Nkangala District, South Africa, concluded that being unbooked was found to predict the occurrence of BBAs (4). The findings of these studies were consistent with the findings of the present study. Although there is no similar evidence from Ethiopia, our findings are supported by the 2019 mini-EDHS report, which revealed that, compared with 14% of births to mothers with no ANC visits, 74 % of births to mothers who attended four or more ANC visits were delivered in a health facility (7). This finding signals that the ANC program, if properly delivered, will be important for reducing birth before arrival at health care facilities and, subsequently, its adverse perinatal outcomes.

Compared with autonomous women, dependent women had three times greater odds of giving birth before arriving at a health care facility, as per the current study. Similarly, one study showed that women’s autonomy was positively associated with health facility delivery in Ethiopia (30). Another study revealed the strongest association between delivery at healthcare facilities attended by skilled birth attendants in the southern African region among women who made decisions on household income solely (31). Women’s autonomy can influence their decision to seek health care and could result in delays in reaching a health care facility to give birth as early as possible if they are not adequately autonomous. Therefore, this results in birth before arrival. We require policy actions that increase women’s autonomy at home, which could be effective in helping assure women’s delivery at health care facilities.

Furthermore, the current study revealed that those women who were not knowledgeable about labor symptoms were two times more likely to experience birth before arrival than those who were knowledgeable. Our finding was supported by one study from Tharaka Nithi County of Kenya, which reported that the obstetric risk factors associated with BBA were recognition of the onset of labor and knowledge of signs and symptoms of labor (23). This finding implies that having poor knowledge of labor symptoms could affect the ability of women to recognize the onset of labor early and seek healthcare facility delivery early. Therefore, standard guidelines for ANC in Ethiopia are needed and emphasize that every pregnant woman should receive ANC from a skilled provider (7), which must include counseling and personal education with women concerning labor symptoms.

Additionally, women who were prepared for the index pregnancy were less likely to experience birth before arrival than women with no birth preparedness plan, as per the current study. Similarly, one study from Tharaka Nithi County, Kenya, reported that the obstetric risk factors associated with BBA were the identification of healthcare facilities for delivery, the identification of means of transport, financial preparation for hospital delivery, and basic supplies for birth (23). These are the components of the birth preparedness plan that were revealed by the Kenya study, and these were used in our study as constructs to measure and compute variables for birth preparedness. As Ethiopia has adopted the WHO’s goal-oriented focused antenatal care for promoting the health and survival of mothers and babies, one of its basic components, which includes an individualized birth plan, complication readiness, and emergency preparedness, is expected to alleviate the problem of BBA if properly performed. Having first and second delays, status of the road, time of delivery, and income were unrelated to the probability of birth before arrival.

### Strengths and limitations of the study

Our study revealed that the delivery of babies occurred between home and health care facilities and revealed the prevalence of BBA and its associated factors, as this issue has received less attention in the literature in Ethiopia. The study has a limitation in that it was based on a cross-sectional design due to its unclear prevalence of BBA, but we recommend that it be based on a community-based case–control study. The results obtained from the current study were based on interviews with women and cannot be free from recall and social desirability biases.

## Conclusion and recommendations

This study assessed the prevalence of birth before arrival and its associated factors among postpartum women in southern Ethiopia. The prevalence of birth before arrival among women who gave birth in the last 6 months preceding this study in the study area was unacceptably high. A statistically significant association was observed between birth before arrival and having no antenatal care, dependent women’s autonomy status, being not knowledgeable about labor symptoms, and having birth preparedness toward the index birth. Intervening in preventing birth before arriving through an effective antenatal acre program, especially focusing on individualized counseling concerning knowledge of labor symptoms and birth preparedness plans and providing extra vigilant attention to enhancing women’s autonomy in the community, may help reduce the number of births before arriving and adverse perinatal outcomes. Furthermore, more comprehensive study at national level should be done in the future.

## Data Availability

The original contributions presented in the study are included in the article/supplementary material, further inquiries can be directed to the corresponding author.
